# Supplementary material for: The Potential of Phaeodactylum as a Natural Source of Antioxidants for Fish Oil Stabilization
Source: Foods. 2022 May 18;11(10):1461. doi: 10.3390/foods11101461 (PMC9140547; doi:10.3390/foods11101461)
Supplement: Supplementary file 1 [file foods-11-01461-s001.zip › foods-1682359-supplementary.pdf]

Supplementary Materials

This file provides information on statistical significance supplementary to Figures 2–5.

Table S1: Statistical significance supplementary to Figure 2: Primary Oxidation (Part I). Different letters indicate statistical differences with $\alpha = 0.05$ within each system ('Fish oil' and 'Fish oil + *Phaeodactylum* biomass (2.5%)').

	**Day 0**	**Day 1**	**Day 3**	**Day 5**	**Day 8**	**Day 14**	**Day 21**
Fish oil	E	DE	D	C	BC	A	B
Fish oil + *Phaeodactylum* biomass (2.5%)	D	CD	CD	BCD	AB	A	ABC

Table S2: Statistical significance supplementary to Figure 3: Secondary Oxidation (Part I). Different letters indicate statistical differences with $\alpha = 0.05$ within each system ('Fish oil' and 'Fish oil + Phaeodactylum biomass (2.5%)').

**(E,E)-2,4-Heptadienal (Figure 3a)**	**Day 0**	**Day 1**	**Day 3**	**Day 5**	**Day 8**	**Day 14**	**Day 21**
Fish oil	B	AB	AB	AB	AB	AB	A
Fish oil + *Phaeodactylum* biomass (2.5%)	B	B	B	B	B	A	A
**(E)-2-Pentenal (Figure 3b)**	**Day 0**	**Day 1**	**Day 3**	**Day 5**	**Day 8**	**Day 14**	**Day 21**
Fish oil	B	AB	AB	AB	AB	AB	A
Fish oil + *Phaeodactylum* biomass (2.5%)	C	C	C	C	C	B	A
**(Z)-2-Butenal (Figure 3c)**	**Day 0**	**Day 1**	**Day 3**	**Day 5**	**Day 8**	**Day 14**	**Day 21**
Fish oil	A	A	A	A	A	A	A
Fish oil + *Phaeodactylum* biomass (2.5%)	B	B	B	B	B	A	A

Table S3: Statistical significance supplementary to Figure 4: Primary Oxidation (Part II). Different letters indicate statistical differences with $\alpha = 0.05$ within each system ('Pure Phaeodactylum total lipid extract', 'Mixture 79/21', 'Mixture 49/51', 'Mixture 6/94', 'Mixture 1/99', Pure Fish oil). The last three systems were only analyzed until day 28 since they already showed clear oxidation.

	**Day 0**	**Day 4**	**Day 8**	**Day 12**	**Day 20**	**Day 28**	**Day 42**	**Day 56**	**Day 70**
Pure *Phaeodactylum* total lipid extract	A	A	A	A	A	A	A	A	A
Mixture 79/21	A	A	A	A	A	A	A	A	A
Mixture 49/51	A	A	A	A	A	A	A	A	A
Mixture 6/94	B	A	B	B	B	B	-	-	-
Mixture 1/99	B	A	B	B	B	B	-	-	-
Pure Fish oil	C	A	B	BC	BC	BC	-	-	-

Table S4: Statistical significance supplementary to Figure 5: Secondary Oxidation (Part II). Different letters indicate statistical differences with α = 0.05 within each system ('Pure *Phaeodactylum* total lipid extract', 'Mixture 79/21', 'Mixture 49/51', 'Mixture 6/94', 'Mixture 1/99', Pure Fish oil). The last three systems were only analyzed until day 28 since they already showed clear oxidation.

**(E,E)-2,4-Heptadienal (Figure 4a,b)**	**Day 0**	**Day 4**	**Day 8**	**Day 12**	**Day 20**	**Day 28**	**Day 42**	**Day 56**	**Day 70**
Pure *Phaeodactylum* total lipid extract	A	C	C	BC	B	B	C	BC	BC
Mixture 79/21	A	C	C	C	C	C	B	B	B
Mixture 49/51	A	C	C	C	C	C	B	B	B
Mixture 6/94	D	CD	BC	AB	A	A	-	-	-
Mixture 1/99	E	D	C	B	A	A	-	-	-
Pure Fish oil	E	D	C	B	A	A	-	-	-
**(E)-2-Pentenal (Figure 4c,d)**	**Day 0**	**Day 4**	**Day 8**	**Day 12**	**Day 20**	**Day 28**	**Day 42**	**Day 56**	**Day 70**
Pure *Phaeodactylum* total lipid extract	A	D	D	D	D	CD	BC	AB	AB
Mixture 79/21	A	C	C	C	C	C	B	B	B
Mixture 49/51	A	C	C	C	C	C	B	AB	AB
Mixture 6/94	B	A	A	A	A	A	-	-	-
Mixture 1/99	B	A	A	A	A	AB	-	-	-
Pure Fish oil	B	AB	AB	A	AB	AB	-	-	-
**(Z)-2-Butenal (Figure 4e,f)**	**Day 0**	**Day 4**	**Day 8**	**Day 12**	**Day 20**	**Day 28**	**Day 42**	**Day 56**	**Day 70**
Pure *Phaeodactylum* total lipid extract	A	A	A	A	A	A	A	A	A
Mixture 79/21	A	A	A	A	A	A	A	A	A
Mixture 49/51	A	A	A	A	A	A	A	A	A
Mixture 6/94	B	A	A	A	A	A	-	-	-
Mixture 1/99	B	A	A	A	A	A	-	-	-
Pure Fish oil	B	A	A	A	A	A	-	-	-
